# The prevalence of hypertension and influencing factors among the employees of a university hospital

**DOI:** 10.4314/ahs.v20i4.24

**Published:** 2020-12

**Authors:** Seher Kurtul, Funda Kaya Ak, Meral Türk

**Affiliations:** 1 Department of Occupational Disease, Ege University Faculty of Medicine, Izmir, Turkey. seherkurtul79@gmail.com; 2 Department of Occupational Disease, Ege University Faculty of Medicine, Izmir, Turkey. funda292kaya@hotmail.com; 3 Department of Occupational Disease, Ege University Faculty of Medicine, Izmir, Turkey. meralturk2010@gmail.com

**Keywords:** Hypertension, hospital, health care worker

## Abstract

**Background:**

Hypertension is a serious disease with increasing worldwide prevalence, leading to life-threatening complications.

**Methods:**

This cross-sectional study was carried out in a university hospital. The Occupational Health and Safety Unit data concerning the health examinations of employees were used to determine the prevalence of hypertension in a university hospital and to define the relationship between hypertension and sociodemographic and occupational parameters. Logistic regression analyses were performed for the variables having a significant association with high blood pressure.

**Results:**

The data generated during the periodic examination of 3,480 (92%) of all employees (3,780) were analyzed. The prevalence of hypertension was 14.8%. The prevalence of hypertension was found to be 13.5%, 13.9%, and 23.7% among physicians, non-physician healthcare personnel and officers respectively. The logistic regression model revealed a statistically significant correlation between hypertension and male gender, age and BMI.

**Conclusion:**

The prevalence of hypertension was highest among staff members. Special programs would facilitate the diagnosis, control, and prevention of high blood pressure among the high-risk groups, especially men, the elderly and the obese hospital employees.

## Introduction

Hypertension (HT) is a serious disease with increasing worldwide prevalence, leading to life-threatening complications. Hypertension is a silent disease in which the symptoms are rarely seen during the early stages, until a serious complication such as heart attack, stroke or chronic kidney disease occurs.^1^,^2^ In a study evaluating the prevalence of HT at a global level and involving 19.1 million people, it was shown that although there were regional differences, the prevalence of HT increased globally in the last four decades and the number of hypertensive persons increased by 90% to 1.13 billion in 2015.^3^ It is estimated that 7.5 million people die from hypertension-related diseases worldwide, which is responsible for about 12.8% of all deaths.^4^ It is also estimated that approximately 75% of the world's hypertensive population will be in developing countries by 2025.^5^ In addition, the global economic burden created by hypertension is estimated to be approximately 370 billion dollars, which constitutes 10% of all healthcare expenditures.^6^

According to the data from the Turkish Statistical Institute, the prevalence of hypertension among individuals aged 15 or above was 16.1% in 2014; it was the third most common disease.^7^ In the Turkish Hypertension Prevalence study (PatenT-2012), the prevalence of hypertension among individuals over 18 years of age was 30.3% was reported and according to age groups; the prevalence of hypertension, 5% in 18–29 age group, 11.5% in 30–39 age group, 29.7% in 40–49 age group, 53.6% in 50–59 age group, 67.9% in 60–69 age group, 85.2% in 70–79 age group, 76.2% in 80 years and over was found^2^.

Several risk factors are involved in the etiology of hypertension: age, geographic factors, genetics, socio-economics, ethnicity, diet and nutrition. Occupation-related factors are also among the significant risk factors for hypertension.^8^ The prevalence of hypertension has been found to vary among various occupational groups; some studies have reported the prevalence among bankers and police officers as 39.3% and 27%, respectively^9^,^10^ while others reported a prevalence of 26% or 21.3% among hospital employees.^11^,^12^

Healthcare workers are exposed to significant stress-related risk factors such as working overtime, high workload, time-intensive pressures, difficult or complex tasks, inadequate breaks, monotony and poor physical conditions. In addition, during the treatment procedures, they are exposed to various hardships such as long periods of time on their feet, insomnia during night shifts, and dietary irregularities, depending on their workload. Hospitals with many occupational risk factors are categorized as a ‘very dangerous workplace’. Studies investigating the prevalence of hypertension among healthcare workers in Turkey are limited. It is important to define the prevalence of hypertension subgroups of people in various lines of work in order to lay down prevention and treatment plans for hypertension. Therefore, the current study was conducted at the Ege University Faculty of Medicine Hospital, which is the largest hospital of the region, and which has employees in different occupational positions. The aim of this study was to determine the prevalence of hypertension in hospital employees and to define the relationship between hypertension and socio-demographic and occupational characteristics.

## Methods

The study has a descriptive cross-sectional design and included all employees who participated in the annual periodic examination conducted in 2017 by the Occupational Health and Safety unit of the Ege University School of Medicine Hospital. The required ethical approval for this study was obtained from the local ethics committee of the hospital (20.03.2018, No: 18-3.1 /30). The largest university hospital in the Aegean Regon, it has a bed capacity of 2000 and provides outpatient services to approximately one million patients annually. The hospital employed a total of 3,780 personnel including doctors, nurses, laboratory staff, pharmacists, technicians, administrative staff and other assisting personnel. Rather than sampling a portion of the population, the study included all of the 3,480 employees who underwent the periodical examination in 2017, with a coverage rate of 92%.

## Methods

During 2017 (January-December), employees were invited in order to an annual periodic examination. The examinations and measurements were performed by two physicians and two nurses in the occupational health and safety unit. After the annual periodic examination, blood pressure and anthropometric measurements were done. The health service workers were trained in standardized blood pressure measurement and anthropometric measurement protocols.

The data was obtained from the anamnesis form, including various data on socio-demographic characteristics, educational status, employment type, monthly work hours, position, years of service, hospital unit served in, shift, etc. After the interview, weight and height measurements were done to calculate the body mass index (BMI). The height measurement was done without shoes, with the heels touching the wall, the back in a straight position, and the head in the normal anatomical position. The weight measurement was done after removing excess clothing and shoes. BMI was calculated by dividing the weight in kilograms (kg) by squared height in meters (m^2^). Subjects were categorized based on their BMI as normal weight (BMI <25 kg/m^2^), overweight (25 ≤BMI<30 kg/m^2^), and obese (BMI ≥30 kg/m^2^).

Blood pressure measurements were performed with a digital monitor (Braun ExactFit ™ 3 - BP6000) using a sleeve suitable for the person's arm circumference. Calibration of the sphygmomanometer is performed at least every six months by comparison with mercury manometers. During the 30-minute period before the measurement, it was ensured that the employee did not smoke, drink tea or coffee and take caffeine. The systolic/diastolic blood pressure measurements were done twice on the right arm of the subject after rsting in the sitting position for at least ten minutes by the physician who performed the physical examination; the arithmetic mean of these two measurements was used. Those who had been diagnosed with hypertension and taking antihypertensive medication or those who had systolic blood pressure ≥140 mmHg and/or diastolic blood pressure ≥90 mmHg were considered hypertensive.^13^ Employees were categorized into four groups based on their positions: a) physicians, b) non-physician healthcare personnel (nurses, technicians, laboratory staff, pharmacists and dieticians), c) administrative staff and d) others.

## Statistical analysis

Data analysis was performed with SPSS v18.0 program (SPPSS Inc., Chicago, USA). In descriptive statistics, categorical variables were presented as number and percentage (%); numerical variables were presented as mean ±standard deviation or median (minimum and maximum). Chi-square test was used to compare independent categorical variables. Student's t-test or Mann-Whitney U test was used in two-group comparisons of numerical variables; one-way variance analysis (One-Way ANOVA) or Kruskal-Wallis test was used for multi-group comparisons of numerical variables. A logistic regression model was used for multivariate analyses. The statistical significance level was set as P <0.05.

## Results

The study included a total of 3,480 subjects; 2,315 (66.5%) were women and 1,165 (33.5%) were men. The average age was 39.8 ± 10 years (38.76 ± 9.32 years for women and 41.89 ± 11.17 years for men). Demographic data and educational status were shown in Table 1. A significant difference was observed between the prevalence of hypertension among men and women (P <0.05). The prevalence of hypertension was highest in the group aged 60 years or above (P <0.001). The majority of the workers were married, and hypertension was more frequent among these subjects (P <0.001). Of the subjects, 78.7% held a Bachelor's degree or higher, 10.2% had an associate degree, and 11.1% had a high school degree or below. Hypertension was most frequently observed in the group with a high school degree or below (P <0.001). The prevalence of smoking was 35%; that of alcohol consumption was 28.3%. A significant relationship was found between having high blood pressure and smoking or alcohol consumption (P <0.01). A majority of the workers (48.9%) had BMI<25; the frequency of hypertension increased as the BMI increased (P <0.001).

**Table 1:** Demographic characteristics of workers and frequency of hypertension

Characteristics	N_total_ (%×)	N_hypertensive_ (%××)	P
Gender			<0.001
Male	1165 (33.5)	270 (23.2)	
Female	2315 (66.5)	245 (10.6)	
Age			<0.001
<30	728 (20.9)	21 (2.9)	
30–39	1041 (29.9)	65 (6.2)	
40–49	1053 (30.3)	186 (17.7)	
50–59	529 (15.2)	176 (33.3)	
≥60	117 (3.4)	67 (57.3)	
Marital Status			<0.001
Single	1070 (30.7)	90 (8.4)	
Married	2410 (69.3)	425 (17.6)	
Education			<0.001
High school or below	387 (11.1)	108 (27.9)	
Associate degree	355 (10.2)	55 (15.5)	
Bachelor's or higher	2738 (78.7)	352 (12.9)	
Smoking			<0.01
Yes	1219 (35)	206 (16.9)	
No	2261 (65)	309 (13.7)	
Alcohol Consumption			<0.01
Yes	984 (28.3)	176 (17.9)	
No	2496 (71.7)	339 (13.6)	
BMI			<0.001
<25	1701 (48.9)	107 (6.3)	
25≤ <30	1254 (36)	239 (19.1)	
≥30	518 (14.9)	169 (32.6)	

× column percentage

×× row percentage

Calculating subjects 40 years of age and above, the number within each category is as follows: 57.2% of males and 44.9% of females; 26.8% of single and 58.9% of married subjects; 41% of physicians, 35.3% of nurses, 76% of administrative staff, 61.9% of technicians, 43% of those working in non-surgical branches, 41.9% of those in surgical branches, 65.9% of those in the basic medical branches, 76.9% of the employees in the administrative units, and 68.3% of the employees in the other units; 63.4% of those working 160 hours or less per month and 31.6% of those working 160 hours or more per month; and finally, 54.2% of permanent employees and 2.9% of the employees on contract. The distribution of age groups according to the socio-demographic and professional characteristics is shown in Table 2.

**Table 2:** Distribution of age groups according to socio-demographic and professional characteristics

Age, N (%)	<30	30–39	40–49	50–59	≥60	Total
Gender						
Male	494(%21.4)	779(%33.7)	724(%31.3)	265(%11.5)	48(%2.1)	2310(%100)
Female	234(%20.2)	262(%22.6)	329(%28.4)	264(%22.8)	69(%6)	1158(%100)
Marital Status						
Single	486(%45.8)	291(%27.4)	183(%17.2)	85(%8)	17(%1.6)	1062(%100)
Married	242(%10.1)	750(%31.2)	870(%36.2)	444(%18.5)	100(%4.2)	2406(%100)
Position						
Physician	383(%35.2)	258(%23.7)	207(%19)	173(%15.9)	66(%6.1)	1087(%100)
Nurse	295(%24.8)	475(%39.9)	317(%26.6)	90(%7.6)	13(%1.1)	1190(%100)
Admin. staff	2(%0.5)	93(%23.4)	193(%48.6)	103(%25.9)	6(%1.5)	397(%100)
Technician	41(%7.9)	157(%30.1)	220(%42.2)	81(%15.5)	22(%4.2)	521(%100)
Unit						
Non-surgical	443(%26.1)	524(%30.9)	445(%26.2)	234(%14)	47(%2.8)	1697(%100)
Surgical	250(%25)	332(%33.2)	280(%28)	104(%10.4)	35(%3.5)	1001(%100)
Basic medical sci.	28(%8.8)	81(%25.4)	117(%36.7)	80(%25.1)	13(%4.1)	319(%100)
Administrative unit	4(%1)	90(%22.1)	197(%48.4)	96(%23.6)	20(%4.9)	407(%100)
Other	4(%6.3)	16(%25.4)	26(%41.3)	15(%23.8)	2(%3.2)	63(%100)
Monthly Work Hour						
≤160 hours	248(%13.1)	444(%23.5)	665(%35.2)	429(%22.7)	104(%5.5)	1890(%100)
>160 hours	471(%30.6)	583(%37.8)	381(%24.7)	95(%6.2)	11(%0.7)	1541(%100)
Type of Employment						
Permanent	476(%15.3)	951(%30.5)	1045(%33.5)	527(%16.9)	117(%3.8)	3116(%100)
On-contract	252(%71.6)	90(%25.6)	8(%2.3)	2(%0.6)	0	352(%100)

Of the participatin g hospital employees, 56.4% were physicians, 31.5% were non-physician workers (nurses, technicians, laboratory personnel, pharmacists and dieticians), 11.4% were administrative staff members and 0.7% were other personnel (cleaning staff, drivers and engineers). A significant relationship was found between the positions of the employees and the prevalence of hypertension (P <0.001). The prevalence of hypertension was higher among administrative staff members. Of the participating hospital employees, 49% were in non-surgical branches, 28.9% in surgical branches, 12.2% in administrative departments, and 10% in basic medical branches. The prevalence of hypertension was higher among those in the administrative positions, which was consistent with the results given above (P <0.001). With respect to the duration of current work position, the largest group was those who worked for 1–10 years (43.4%). The rate of hypertension increased as the number of years in the current work position increased (P <0.001). Among the personnel who did not work in shifts (91.1%) and those who did not work night shifts (61.5%), the rate of hypertension was significantly higher (P <0.001 for each). The frequency of hypertension was higher in employees working for 160 hours or less per month (P <0.001). A great majority of the employees (89.7%) were in permanent positions while the remaining 10.3% were on contract; hypertension was significantly more frequent in the former group (P <0.001). The prevalence of hypertension with respect to occupationalharacteristics is shown in Table 3.

**Table 3:** Prevalence of hypertension by occupational characteristics

Characteristics	N_total_ (%×)	N_hypertensive_ (%××)	P
Position			<0.001
Physician	1962(56.4)	264(%13.5)	
Non-physician care	1097(31.5)	152(%13.9)	
Staff	397(11.4)	94(%23.7)	
Other	24(0.7)	5(%20.8)	
Unit			<0.001
Non-surgical	1685(48.4)	213(%12.6)	
Surgical	1006(28.9)	138(%13.7)	
Basic medical sci.	319(9.2)	61(%19.1)	
Administrative unit	407(11.7)	97(%23.8)	
Other	63(1.8)	6(%9.5)	
Years of Service			<0.001
1–10	1511(43.4)	82(%5.4)	
11–20	895(25.7)	124(%13.9)	
21–30	784(22.5)	202(%25.8)	
31–40	222(6.4)	81(%36.5)	
41 and above	18(0.5)	12(%66.7)	
Shift Work			<0.001
Yes	311(8.9)	25(%8)	
No	3169(91.1)	490(%15.5)	
Night Shift			<0.001
Yes	1341(38.5)	96(%7.2)	
No	2139(61.5)	419(%19.6)	
Monthly Work Hour			<0.001
≤160 hours	1897(54.5)	373(%19.7)	
>160 hours	1546(44.4)	140(%9.1)	
Type of Employment			<0.001
Permanent	3121(89.7)	496(%15.9)	
On-contract	359(10.3)	19(%5.3)	

× column percentage

×× row percentage

The prevalence of hypertension was found to be 14.8% in our study. Of these, 65.8% consisted of patients who answered yes to the question of whether they had hypertension; the remaining 34.2% were not aware of their hypertension but had systolic blood pressure ≥140 mmHg and/or diastolic blood pressure ≥90 mmHg. Of the subjects with a history of hypertension, 88.2% were using antihypertensive drugs.

In order to determine the most important work-related factors significantly influencing the prevalence of hypertension, a logistic regression analysis was performed on employment variables such as gender, marital status, age, education level, smoking, alcohol use, BMI, position, unit, shift work, night work, monthly working hours and type of employment (Table 4). Among the variables included in the model, being male, being 60 years old or older, and having high BMI were found to be independent risk factors for hypertension. Hypertension was found to be 1.6 times higher in men than women (odds ratio (OR) = 1.621). The risk of being hypertensive was 2 times higher in overweight cases (OR = 2.091) and 4.3 times higher in obese cases (OR = 4.338) compared to those with normal weight. The risk of being hypertensive was found to be significantly increased (9.1 times) with increasing age (OR = 1.091).

**Table 4:** Logistic regression analysis of the risk factors for hypertension

Risk Factors	Estimated Parameter	Standard Error	Odds Ratio	Confidence Interval (%95)	P
Gender					
Male	0.483	0.123	1.621	1.272 – 2.064	<0.000
BMI					
25≤ <30	0.738	0.136	2.091	1.602 – 2.731	<0.000
≥30	1.467	0.154	4.338	3.209 – 5.864	<0.000
Age	0.87	0.10	1.091	1.071 – 1.112	<0.000

## Discussion

In our study, the prevalence of hypertension among hospital employees participating in this study was 14.8%, which was lower than Brazil, higher than the hospital employees in Spain^11^,^14^. This prevalence of hypertension among hospital employees was similar to general population of Turkey^2^,^7^. Based on 2014 data from the Turkish Statistical Institute the prevalence of hypertension among individuals aged 15 or older was 16.1% in Turkey; in another study involving individuals aged 18 or older, the rate was 30.3%.^2^,^7^ In studies from Asian countries, the prevalence was found to be 33.7% (South Korea) and 27.8% (Malaysia).^15^,^16^ The average prevalence was 44% in some European countries, and 28%n North American countries.^17^

Studies have shown that the incidence of hypertension increases with age and this increase is more prominent in patients over the age of 40.^18^ In this study, one half of the hospital workers were over the age of 40 and age was among the independent predictors of hypertension in the logistic regression analysis. When evaluated according to age groups, hypertension was found in one in six people in the age group 40–49 and in one in three individuals in the age group 50–59. Since the incidence of hypertension is significantly increased among individuals over 40 years of age, they should be considered a priority for hypertension screening.

The prevalence of hypertension was significantly higher in males (23.2%) than females (10.6%), and age was found to be a significant risk factor in the logistic regression analysis, as mentioned previously. Consistent with our findings, the prevalence of hypertension was also higher among males in studies conducted in Greece, Brazil and India.^19^,^11^,^20^ In our study, the prevalence of hypertension in women increased with age and was found to be higher than men. This is thought to be related to changes in estrogen and progesterone levels after menopause.^21^ In our study, 26.8% of the single individuals and 58.9% of those who were married were over the age of 40. The mechanisms underlying the effect of marital status on hypertension are not entirely understood. Some explanations have been suggested for effects of marital status including psychopathological factors, neuroendocrine pathways, health behaviors, biological mediators and immune pathways.^22^ Similar to the study by Onwuchekwa et al.^23^, a lower prevalence of hypertension was found for those who were not married in our study.

Education level is related to socio-economic level and affects lifestyle, nutrition and physical activity. It has been reported that the incidence of hypertension decreases as education level increases.^11^ In our study which mostly consisted of university graduates, the prevalence of hypertension decreased as the education level increased. Even though the level of education was not found to be a significant risk factor in the logistic analysis, it should be taken into account due to the difference in the frequency of hypertension among individuals with different educational levels. In our study, one out of three employees had a history of smoking and drinking alcohol. The smoking rate we found was close to that of other hospital employees'. As a result of vasoconstriction caused by the sympathomimetic effect of nicotine and other substances in cigarettes, an increase in blood pressure occurs. Thankappan et al.^24^ found that smokers carried 1.3 times higher risk of hypertension while those who used alcohol had 1.23 times higher risk. Similarly, hypertension was significantly more frequent in patients with a history of smoking and alcohol use.

In this study, the risk of hypertension was 2 times higher in overweight and 4.3 times higher in obese individuals compared to those with normal weight. More than half of the employees had increased BMI, which was thought to be due to an unhealthy lifestyle. Health workers, due to work intensity, work stress, night work, shift work eat more irregularly and do exercise less. Body mass index increases with age. Similar to the findings of Rampal et al.^16^, the number of hypertensive individuals was found to increase with increasing BMI. When the employees were evaluated according to their occupational positions and the units in which they work, the prevalence of hypertension was found significantly higher among the staff members and employees working in administrative units. The facts that staff members were mostly associated with administrative units and that 76% of them were over the age of 40 were considered to be responsible for higher rates of hypertension. In addition to socio-demographic parameters, stress factors that increase the risk of hypertension up to 80% including decision-making, lack of authority, excessive psychological demand, overwork and short deadlines, which were found to influence the differences in hypertension rates between units.^25^ In a previous study, work-related, long-term mental stress was found to be associated with hypertension in white-collar workers.^24^ Administrative units are more legislative and hierarchical than the medical units and staff members cannot decide freely, so they work under more stress. Hypertension rate was found to be higher among physicians (13.9%) compared to nurses (9.3%). According to the age groups, physicians were older than nurses. Furthermore, studies from developed countries found that a significant number of physicians were not registered with a family physician and did not individually follow current preventive health guidelines.^26^–^28^

Although there are some studies indicating that the lack of job security is a risk factor for hypertension, hypertension was observed more frequently in employees with permanent status in our study. The on-contracted employees consisted of young workers. The fact that only 2.9% of the contract employees were above the age of 40 might have contributed to this result.^29^,^30^

In our study, hypertension was less frequent among those working in shifts and those working at night. Working in shifts is thought to cause hypertension by disturbing the biological rhythm. However, previous studies reported conflicting findings with regard to the relationship between working in shifts and frequency of hypertension. Similar to our findings; Sfreddo and colleagues reported that there was no significant relationship between working in shifts and blood pressure level in nurses.^31^ In our study, it was found that hypertension was more common in subjects who worked more than 160 hours a month on average. Different views have been reported in studies investigating the relationship between work hours and hypertension.^32^–^34^. In our hospital, the group working the most at night and working in shifts is the assistant doctors and nurses who are starting to work in the young age group. Therefore, as age increases, the number of shift work, night work and total monthly working hours decreases and this seems to be consistent with our results.

Although the relationship between various occupation-related factors and the prevalence of hypertension were found to be statistically significant, logistic regression analysis indicated a stronger influence of variables such as gender, age, and BMI on the probability of developing hypertension.

There are some limitations to this study. First, blood pressure was measured on one day only. For this reason, the prevalence of hypertension may be overestimated due to high blood pressure at the time of measurement. However, many of the studies of this nature do not take blood pressure measurements several times. A second limitation is that it is likely that the diagnosis of those with hypertension may be inaccurate.

## Conclusion

This study reveals the influence of socio-demographic and occupation-related factors on blood pressure, underlining the significant problem of hypertension and the need for programs to prevent and control hypertension in healthcare workers in Turkey. The results indicate that there is a need for special programs targeted at high-risk groups such as men, the elderly and obese individuals. These special programs should aim at adopting healthy lifestyles (implementation of healthy eating and physical activity programs), taking measures to prevent high blood pressure, facilitating early diagnosis of the disease and increasing awareness about hypertension to ensure adequate control.
